# Chemical–Genetic Profiling of Imidazo[1,2-*a*]pyridines and -Pyrimidines Reveals Target Pathways Conserved between Yeast and Human Cells

**DOI:** 10.1371/journal.pgen.1000284

**Published:** 2008-11-28

**Authors:** Lisa Yu, Andres Lopez, Abderrahmane Anaflous, Brahim El Bali, Abdellah Hamal, Elke Ericson, Lawrence E. Heisler, Angus McQuibban, Guri Giaever, Corey Nislow, Charles Boone, Grant W. Brown, Mohammed Bellaoui

**Affiliations:** 1 Department of Biochemistry, University of Toronto, Toronto, Ontario, Canada; 2 Terrence Donnelly Centre for Cellular and Biomolecular Research, University of Toronto, Toronto, Ontario, Canada; 3 Banting and Best Department of Medical Research, University of Toronto, Toronto, Ontario, Canada; 4 Department of Molecular Genetics, University of Toronto, Toronto, Ontario, Canada; 5 Laboratoire de Chimie du Solide Minéral et Analytique, Département de Chimie, Faculté des Sciences, Université Mohamed Premier, Oujda, Morocco; 6 Laboratoire de Génétique et Biotechnologies, Département de Biologie, Faculté des Sciences, Université Mohamed Premier, Oujda, Morocco; 7 Faculty of Pharmacy, University of Toronto, Toronto, Ontario, Canada; 8 Faculté Pluridisciplinaire de Nador, Université Mohamed Premier, Nador, Morocco; Yale University, United States of America

## Abstract

Small molecules have been shown to be potent and selective probes to understand cell physiology. Here, we show that imidazo[1,2-*a*]pyridines and imidazo[1,2-*a*]pyrimidines compose a class of compounds that target essential, conserved cellular processes. Using validated chemogenomic assays in *Saccharomyces cerevisiae*, we discovered that two closely related compounds, an imidazo[1,2-*a*]pyridine and -pyrimidine that differ by a single atom, have distinctly different mechanisms of action in vivo. 2-phenyl-3-nitroso-imidazo[1,2-*a*]pyridine was toxic to yeast strains with defects in electron transport and mitochondrial functions and caused mitochondrial fragmentation, suggesting that compound 13 acts by disrupting mitochondria. By contrast, 2-phenyl-3-nitroso-imidazo[1,2-*a*]pyrimidine acted as a DNA poison, causing damage to the nuclear DNA and inducing mutagenesis. We compared compound 15 to known chemotherapeutics and found resistance required intact DNA repair pathways. Thus, subtle changes in the structure of imidazo-pyridines and -pyrimidines dramatically alter both the intracellular targeting of these compounds and their effects in vivo. Of particular interest, these different modes of action were evident in experiments on human cells, suggesting that chemical–genetic profiles obtained in yeast are recapitulated in cultured cells, indicating that our observations in yeast can: (1) be leveraged to determine mechanism of action in mammalian cells and (2) suggest novel structure–activity relationships.

## Author Summary

We have shown that chemical–genetic screening allows structure–activity studies of chemical compounds at a very high resolution. In analyzing the effects of closely related imidazo-pyridine and -pyrimidine compounds, we found two compounds that likely act as oxidizing agents, yet target different organelles. The imidazo-pyridine affected mitochondrial functions whereas the imidazo-pyrimidine caused nuclear DNA damage. Remarkably, the only difference between these two compounds is the presence of a nitrogen atom at position 8. Thus, in addition to demonstrating the potential for high resolution in chemical–genetic studies, our work suggests that subtle changes in compound chemistry can be exploited to target different intracellular compartments with very different biological effects. Finally, we show that chemical–genetic profiling in yeast can be used to infer mode of action in mammalian cells. The specificity of compound 15 in eliciting a nuclear DNA damage response in evolutionarily diverse eukaryotes suggests that it will be of great utility in studying the cellular response to nuclear oxidative damage.

## Introduction

Chemical-genetic technologies in the baker's yeast *Saccharomyces cerevisiae* have proven to be a powerful means to study the mode of action of biologically active compounds (reviewed in [1],[2]). When performed on a whole-genome scale, these methodologies typically rely on the collection of yeast gene deletion mutant strains [3], comparing the growth of each gene deletion strain to a wild type strain in the presence and absence of compound. The throughput of the technology was improved by applying a competitive growth strategy, in which a pool of all deletion mutants is grown in the presence and absence of compound [4]–[7]. Following growth, deletion strains that are under-represented, and therefore sensitive, in the presence of compound relative to the control condition are identified by hybridizing the unique DNA sequences which flank each deletion to their complements on a microarray [3]. Compounds can be grouped according to the similarities of their chemo-genomic profiles, which is the total spectrum of gene deletions that result in sensitivity to a given compound, to reveal similarities in the biological responses to query compounds [8]–[11]. Chemical-genetic profiling has proven useful in the identification of targets of a variety of different compounds in yeast [4],[5],[8],[10]. Classic studies, including those indicating conservation of rapamycin and FK-506 targets between mammalian and yeast cells [12]–[14], encourage the view that chemical-genetic profiles derived in yeast can reflect mode of action in human cells.

In this study we sought to provide molecular insight into the biological activity of a group of imidazo[1,2-*a*]pyridines (IP) and imidazo[1,2-*a*]pyrimidines (IPM). Members of this family of compounds have been widely used in medicinal chemistry and include pharmacological agents such as the phosphodiesterase 3 inhibitor olprinone [15], the hypnotic zolpidem [16], and the anxiolytic divaplon [17]. Members also exhibit analgesic, anti-inflammatory [18], anti-viral [19],[20], and anti-microbial activities [21]–[24], and have been optimized as cyclin-dependent kinase inhibitors [25] and as GABA receptor ligands [26],[27]. Thus, these compounds appear to target diverse cellular processes.

Previous studies have indicated that changes in the functional group at position 3 and the atom at position 8 have varying effects on antibacterial activity [21],[22],[24]. Here we investigate the properties of eight imidazo[1,2-*a*]pyridines and imidazo[1,2-*a*]pyrimidines. Only the 3-nitroso derivatives had significant bioactivity in yeast. Chemical-genetic profiling revealed that the atom at position 8 had a dramatic effect on the mode of action of these compounds. 3-nitroso-imidazo[1,2-*a*]pyridine compromised mitochondrial integrity and function whereas 3-nitroso-imidazo[1,2-*a*]pyrimidine caused nuclear DNA damage. Of particular interest, these differences were recapitulated in human cells, suggesting that the underlying mechanisms of action are conserved. The analysis of these two compounds illustrates the power of chemical-genetic screens in predicting the mode of action of chemical compounds, and demonstrates that chemical-genetic profiles from yeast can be used to gain insight into mode of action in human cells.

## Results

### Antifungal Activity of Imidazo[1,2-a]pyridines and Imidazo[1,2-a]pyrimidines

**Figure 1:**
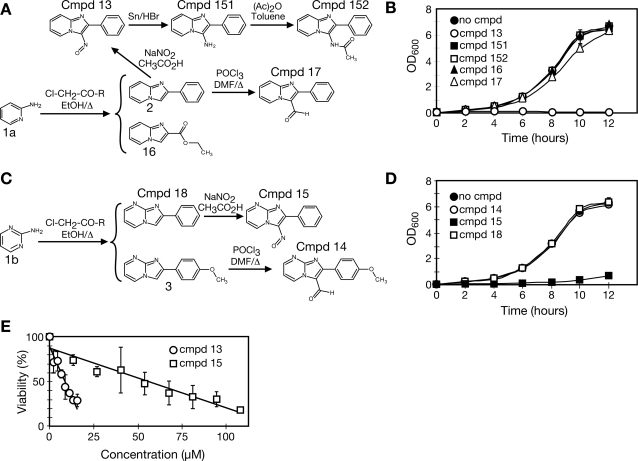
Yeast bioactivity of imidazo-pyridine and imidazo-pyrimidine compounds. A. Structures and synthesis of the imidazo-pyridine compounds analyzed. B. Compound 13 inhibits yeast growth. Cultures of *S. cerevisiae* were grown in the presence of 50 µM of the imidazo-pyridine compounds. Optical density was measured every 2 hours to follow cell growth. C. Structures and synthesis of the imidazo-pyrimidine compounds analyzed. D. Compound 15 inhibits yeast growth. Cultures of *S. cerevisiae* were grown in the presence of 50 µM of the imidazo-pyrimidine compounds. Optical density was measured every 2 hours to follow cell growth. E. EC_50_ determination for compound 13 and compound 15. Cells were treated with the indicated concentrations of the respective compounds for two hours and plated on rich media to determine colony-forming units. Viability is expressed relative to the untreated (vehicle only) control.

Eight IP or IPM derivatives (Figure 1A) were tested for toxicity against budding yeast (*Saccharomyces cerevisiae*) cells. Cells were cultured in the presence of 50 µM of each compound and assayed for growth inhibition in liquid culture (Figure 1B, D). Only compounds 13 and 15, each bearing a nitroso group at position 3, were toxic to yeast cells at 50 µM, consistent with previous observations that the substituent at position 3 is important for activity of IPMs against pathogenic fungi [21]. Higher concentrations (100 µg/ml; 0.4–0.5 mM) of compounds 14, 16, 17, and 18 did not inhibit growth significantly (Figure S1). Various modifications of the nitroso group or elimination of the benzene ring eliminated anti-fungal activity. Nitroso aromatic compounds are bioactive mainly because they are readily reduced to highly reactive nitro radical anions which activate oxygen [28]. Several lines of evidence suggest that compound 13 and 15 are acting as oxidizing agents in vivo. 2-phenylimidazo[1,2-*a*]pyridin-3-amine (compound 151), which is the reduced form of compound 13, was not active on yeast cells (Figure 1B). Furthermore, the antifungal activity of compound 13 and 15 could be partially suppressed by pre-treating cells to induce intracellular accumulation of reduced glutathione (Figure S2), a protective small molecule that is part of the cellular defense against oxidative damage [29]. Finally, we also found that chemical reduction of compounds 13 and 15 in vitro resulted in their inactivation (data not shown).

Compound 13, the IP, was more potent than compound 15, the IPM (Figure 1E). The concentration at which cells retain 50% viability (EC_50_) for compound 13 was 9 µM, compared to 56 µM for compound 15. Subsequent experiments were performed using the same effective concentration for each compound.

### Chemical–Genetic Screens with Compounds 13 and 15

**Figure 2:**
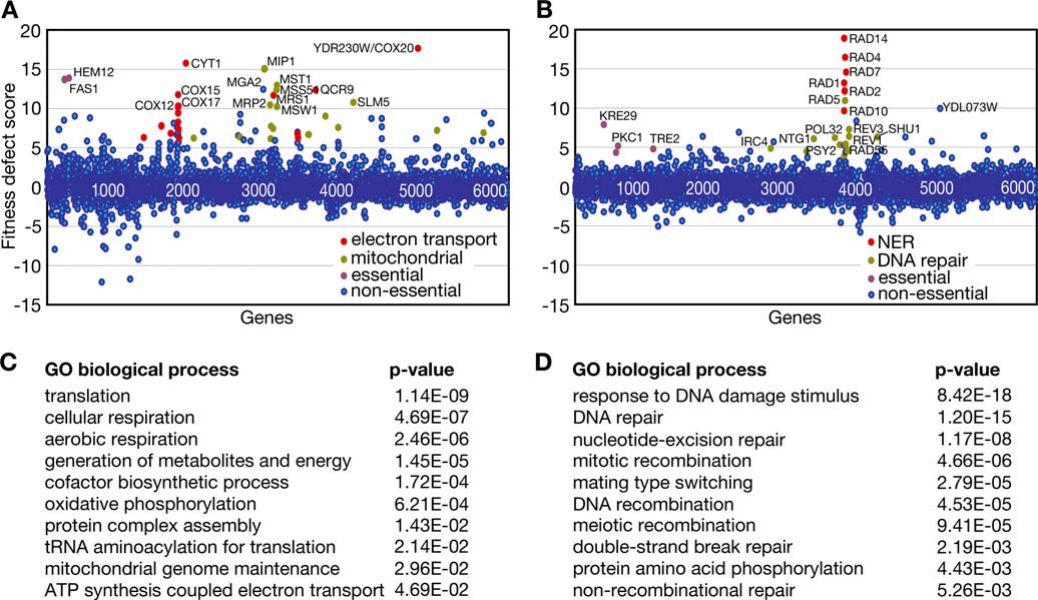
Chemical-genetic profiling of compound 13 and compound 15. A. Identification of gene deletion mutants that confer sensitivity to 9 µM compound 13 by chemical-genetic profiling with the yeast heterozygous essential gene deletion mutants and the homozygous diploid non-essential gene deletion mutants. Fitness defect scores are calculated based on barcode microarray hybridization, and are plotted on the y-axis. Positive values indicate under-representation in the treated pool, and therefore sensitivity of the corresponding mutant. The heterozygous (first 1200 genes) and homozygous deletion mutants are arranged on the x-axis alphabetically. Essential genes (purple), electron transport genes (red) and genes annotated for mitochondrial function (yellow) in the top 50 hits are indicated. B. Identification of gene deletion mutants that confer sensitivity to 9 µM compound 15. Essential genes (purple), nucleotide excision repair genes (NER; red) and DNA repair genes (yellow) in the top 50 hits are indicated. C. Enrichment of GO biological processes in compound 13 sensitive strains with a fitness defect score greater than 4. D. Enrichment of GO biological processes in compound 15 sensitive strains with a fitness defect score greater than 4.

Chemical-genetic screens have successfully predicted the mechanism of action of a variety of compounds in yeast cells [4]–[6],[8],[10]. To determine the molecular basis for the action of compounds 13 and 15, we used the complete pool of barcoded homozygous and essential heterozygous diploid deletion strains of *S. cerevisiae* to identify gene deletions that confer sensitivity to each compound (Figure 2A, B).

Deletion of genes involved in electron transport and mitochondrial function caused cells to be sensitive to compound 13, illustrating the importance of mitochondrial function in resistance to this compound (Figure 2A). Of the 50 most sensitive gene deletions, 38 (76%) have known roles in the mitochondria. Analysis of genes with a z-score greater than 4 (120 genes) revealed a statistically significant enrichment for Gene Ontology (GO) biological process terms relevant to mitochondrial function (Figure 2C), including cellular respiration, generation of energy, oxidative phosphorylation, and mitochondrial genome maintenance. The most enriched GO process was translation, likely due to the abundance of mitochondrial tRNA synthetase and mitochondrial ribosome gene deletion strains identified as sensitive in the screen.

By contrast, profiling of compound 15 revealed that deletions of DNA repair genes, notably those involved in nucleotide excision repair (NER), post replication repair (PRR), and homologous recombination (HR) conferred sensitivity to this compound (Figure 2B). Enriched GO biological processes in the top hits (z-score>4; 45 genes) included response to DNA damage, DNA repair, nucleotide excision repair, and DNA recombination (Figure 2D). The observation that NER, PRR, and HR mutants are all sensitive to compound 15 is reminiscent of tolerance of damage caused by cisplatin, which requires all three of these repair pathways [30]–[33]. NER mutants were most sensitive to compound 15, indicating that NER genes are likely to be the first line of defense against damage caused by compound 15. NER is involved in removal of bulky helix-distorting lesions and adducts on DNA [34],[35], which suggests that compound 15 might directly modify DNA in cells to cause this type of damage.

### Hierarchical Clustering of Compound 13 and 15 with a Compendium of Compounds

**Figure 3:**
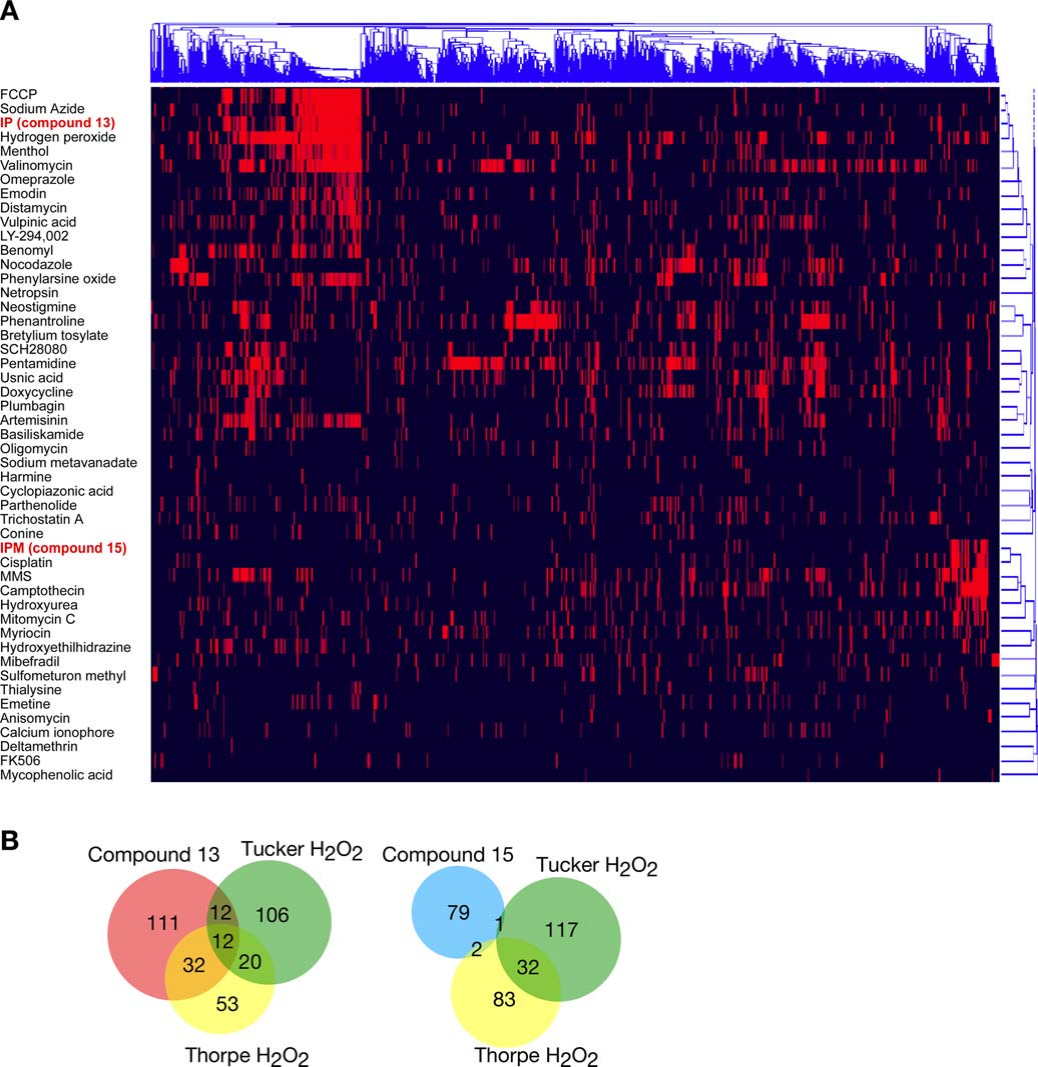
Two-dimensional hierarchical clustering of compounds 13 and 15 with the chemical-genetic profiles of a compendium of 102 compounds and extracts. A. 3418 genes are plotted on the horizontal axis with the gene cluster tree across the top. Compounds are plotted on the vertical axis with the cluster tree on the right. Chemical-genetic interactions are represented as red lines. A portion of the cluster, with 46 compounds, is shown. B. Overlap between the compound 13 and the compound 15 profiles, and the profiles of H_2_O_2_ described in the literature [37],[38]. The Venn diagrams show the number of genes out of a possible 4944 non-essential genes that were identified as being sensitive to compound in each study.

Two-dimensional hierarchical clustering was performed for compound 13 and 15, using a compendium of chemical-genetic profiles [8],[10] obtained for a diverse collection of bioactive compounds (Figure 3A). Chemical-genetic profiles of haploid deletion mutants of non-essential genes were subjected to clustering analysis, in which compounds with similar mechanisms of action cluster together on one axis, and genes with similar spectrums of sensitivities to the compounds cluster together on the other axis.

In agreement with the differences in gene deletions that confer sensitivity to each compound, clustering analysis suggested two very different mechanisms of action for compounds 13 and 15. The profile of compound 13 clustered with carbonyl cyanide para-trifluoromethoxyphenylhydrazone (FCCP), sodium azide, and hydrogen peroxide (Figure 3A). FCCP is a proton ionophore and sodium azide is a respiratory chain inhibitor, suggesting that compound 13 might depolarize the mitochondrial membrane. Compound 13 also clustered with hydrogen peroxide, an oxidizing agent whose tolerance requires functional mitochondria [36]. The genes identified in the compound 13 screen overlapped significantly with those identified in hydrogen peroxide screens reported by Thorpe *et al.* and Tucker and Fields (p = 2.33×10^−36^ and p = 5.91×10^−23^, respectively) [37],[38], further suggesting that compound 13 may be peroxide-like in its character (Figure 3B). The genes identified in the compound 15 screen showed no significant overlap with the reported H_2_O_2_ screens (Figure 3B).

The chemical-genetic profile of compound 15 clustered most closely to cisplatin, which forms both intra-strand and inter-strand DNA crosslinks [39],[40]. It also clustered close to the alkylating agent methyl methanesulfonate (MMS), the topoisomerase inhibitor camptothecin, the replication inhibitor hydroxyurea, and the DNA crosslinking agent mitomycin C, all of which cause DNA damage (Figure 3A). Based on these results, and the enrichment of GO processes evident in the chemical-genetic screens, we hypothesize that compound 13 disrupts mitochondrial function whereas compound 15 causes nuclear DNA damage. Thus despite differing by only a nitrogen atom at position 8, these compounds appear to target different intracellular compartments. Although the mechanism by which this differential targeting occurs remains to be determined, one interesting possibility is that different chemical rearrangements might be responsible for the preferential targeting observed for compounds 13 and 15. Unlike imidazo[1,2-*a*]pyridines, imidazo[1,2-*a*]pyrimidines are susceptible to nucleophilic recyclizations, as the pyrimidine ring makes the system π-deficient [41].

### Compound 13 Disrupts Mitochondrial Function

**Figure 4:**
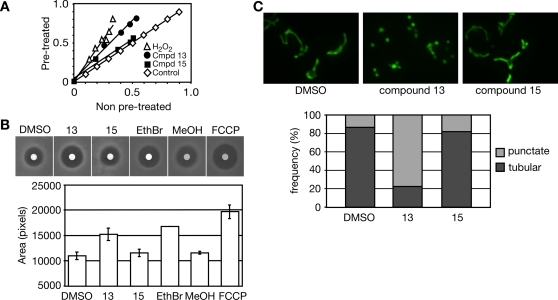
Compound 13 impairs mitochondrial function. A. Pre-treatment with sublethal concentrations of H_2_O_2_ induces tolerance of compound 13. Cells were treated with a sub-lethal (0.25 mM) concentration of H_2_O_2_ for one hour before treatment with higher concentrations of H_2_O_2_ (0 to 3.0 mM, in 1.25-fold increments), compound 13 (0 to 13.4 µM, in 1.25-fold increments), or compound 15. Growth of pre-treated cells relative to untreated cells is plotted. B. Compound 13 causes peroxide sensitivity. Cells were pre-treated with 1% DMSO, 9 µM compound 13, 56 µM compound 15, 25 µM ethidium bromide, 1% MeOH, or 15.7 µM FCCP and spread on YPD plates. Disks containing 6% H_2_O_2_ were placed in the center of each plate and the zone of growth inhibition was measured. The average of two independent experiments is plotted, and error bars span one standard deviation. C. Compound 13 causes mitochondrial fragmentation. Cells were treated with 1% DMSO, 9 µM compound 13, or 56 µM compound 15 for three hours. Mitochondrial morphology was evaluated by fluorescence microscopy, detecting mitochondrial targeted GFP. The fraction of cells displaying the normal tubular mitochondrial morphology and the fraction showing punctate mitochondrial morphology is plotted.

Our chemical-genetic analysis indicated that compound 13 is peroxide-like in character. To determine if hydrogen peroxide and compound 13 induce similar cellular responses, we tested if inducing an oxidative stress response by pretreatment of cells with sub-lethal concentrations of H_2_O_2_ rendered cells resistant to subsequent treatment with compound 13 (Figure 4A). This adaptive response has been observed for H_2_O_2_ in both prokaryotes and eukaryotes [42]–[45]. Cells were pretreated with sub-lethal concentrations of H_2_O_2_ for one hour and then subjected to compound 13, compound 15, or a higher concentration of H_2_O_2_. The adaptive response was measured in liquid growth assays, comparing untreated and H_2_O_2_ pre-treated cells (Figure 4A). A slope of 1.0 indicates that pre-treatment had no effect on the growth rate of cells (Figure 4A, control). Pre-treatment with H_2_O_2_ conferred tolerance to H_2_O_2_ and compound 13, resulting in slopes of greater than 1.0 (Figure 4A). Thus, induction of an oxidative stress response by pre-treatment with H_2_O_2_ results in tolerance to subsequent treatment with compound 13, suggesting that the cellular response to these compounds is similar.

Since H_2_O_2_ tolerance requires functional mitochondria [36], sensitivity to H_2_O_2_ can be used as a proxy measure of mitochondrial function. We tested the effect of pre-treatment of cells with compound 13 on H_2_O_2_ sensitivity (Figure 4B). Cells were treated with compound 13 or 15, or ethidium bromide or the proton ionophore FCCP. Treatment with ethidium bromide or FCCP, which compromise mitochondrial function by different mechanisms, resulted in a greater sensitivity to H_2_O_2_ using halo assays (Figure 4B). Similarly, compound 13 treatment produced a larger clearing than the vehicle (DMSO), suggesting that compound 13 compromises mitochondrial function.

Our accumulated evidence that compound 13 impairs mitochondrial function prompted us to examine whether this compound perturbs mitochondrial morphology (Figure 4C). Yeast cells expressing GFP targeted to the mitochondria were grown in the presence of the vehicle DMSO, compound 13, or compound 15, and mitochondrial morphology was examined by fluorescence microscopy. Upon treatment with DMSO or compound 15 (Figure 4C), most cells exhibited the normal mitochondrial morphology consisting of branched tubular network at the cell cortex [46],[47]. However, 80% of cells treated with compound 13 showed punctate fragmented mitochondria (Figure 4C). Thus, compound 13 caused fragmentation of the mitochondria, a phenotype consistent with impaired mitochondrial function.

### Compound 15 Tolerance Requires DNA Repair Pathways

**Figure 5:**
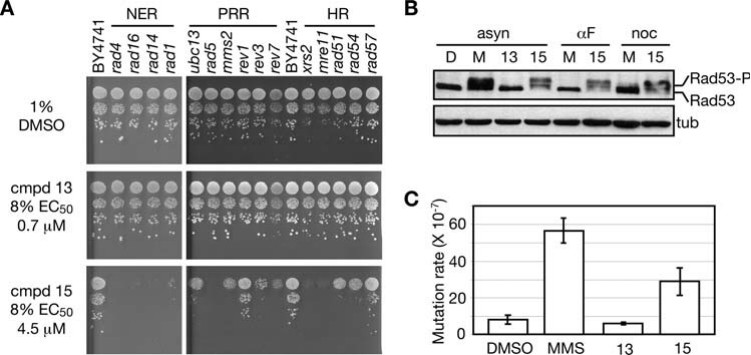
Compound 15 causes nuclear DNA damage in vivo. A. DNA repair mutants are sensitive to compound 15. Ten-fold serial dilutions of the indicated deletion mutant strains were spotted on media containing 1% DMSO, 0.7 µM compound 13 (0.08 EC_50_), or 4.5 µM compound 15 (0.08 EC_50_). Mutants in nucleotide excision repair (NER), postreplication repair (PRR), and homologous recombination repair (HR) are indicated. B. Compound 15 activates the DNA damage checkpoint kinase Rad53. Asynchronous populations of cells (asyn), or cells arrested in G1 (αF) or G2/M (noc), were treated with 56 µM compound 15, or with 0.035% MMS, for 2 hours. Following TCA fixation, cell extracts were fractionated on SDS-PAGE and Rad53 was detected by immunoblot analysis. The effect of the vehicle DMSO (D) is also shown. C. Compound 15 induces mutagenesis. Cells were treated with vehicle (DMSO), 0.035% MMS, 9 µM compound 13, or 56 µM compound 15 for 2 hours. The average mutation rate is plotted, with error bars spanning one standard deviation.

Chemical-genetic analysis indicated that genes involved in three different DNA damage repair pathways are needed for cellular resistance to compound 15. We confirmed the sensitivity of the top hits from the screen with spot dilution assays using individual deletion strains (Figure 5A). Ten-fold serial dilutions of cells were spotted on solid media containing either the vehicle DMSO, compound 13, or compound 15. Since compound 13 and 15 have significantly different potency (Figure 1E), they were used at 8% of their respective EC_50_'s (0.72 µM for compound 13 and 4.45 µM for compound 15). Deletion of genes involved in NER, PRR, and HR conferred sensitivity to compound 15. NER mutants were, in general, most sensitive to compound 15, a strong validation of the chemical-genetic screens. PPR and HR mutants were less sensitive to compound 15, with the upstream genes in each pathway being more sensitive than downstream genes (e.g., *rad5Δ* was the most sensitive PRR mutant, and *xrs2Δ* and *mre11Δ* were the most sensitive HR mutants). Thus, this individual strain analysis not only confirmed the results of the chemical-genetic screens, but also confirmed that tolerance of compound 15 requires DNA repair pathways, whereas tolerance of compound 13 does not.

### Compound 15 Activates the DNA Damage Checkpoint

The DNA damage checkpoint is a signal transduction pathway that is activated in response to DNA damage (reviewed in [48]). We tested whether compound 15 activates the DNA damage checkpoint kinase Rad53 (Figure 5B and Figure S3). The activation of Rad53 by DNA damage is correlated with its phosphorylation, and this results in slower mobility of the protein during SDS-PAGE [49]–[51]. When logarithmic phase cultures were treated with the DNA damaging agent MMS or with compound 15, we observed the characteristic mobility shift of Rad53, indicating checkpoint activation (Figure 5B). Compound 13 did not cause Rad53 activation. We also observed a mobility shift in both the G1 and G2/M arrested cells following treatment with compound 15, whereas MMS did not activate Rad53 in either arrested sample (Figure 5B). We conclude that compound 15 causes checkpoint activation, and since the checkpoint activation does not require passage through S phase it is therefore likely the result of compound 15 causing DNA damage rather than DNA replication stress.

We next assessed the downstream effects of DNA damage caused by compound 15. Since compounds that damage DNA can induce mutagenesis [52],[53], we measured the mutation rate in a wild type strain following treatment with compounds 13 or 15, the DNA alkylating agent MMS, or DMSO, assessing forward mutation to canavanine resistance (Figure 5C). Cells treated with compound 13 had a mutation rate similar to that of DMSO treated cells, while cells treated with compound 15 had an approximately three-fold increase in mutation rate. Thus, compound 15, but not compound 13, induced mutagenesis of nuclear genes, consistent with compound 15 damaging nuclear DNA in vivo.

### The Modes of Action of Compounds 13 and 15 Are Recapitulated in Human Cells

**Figure 6:**
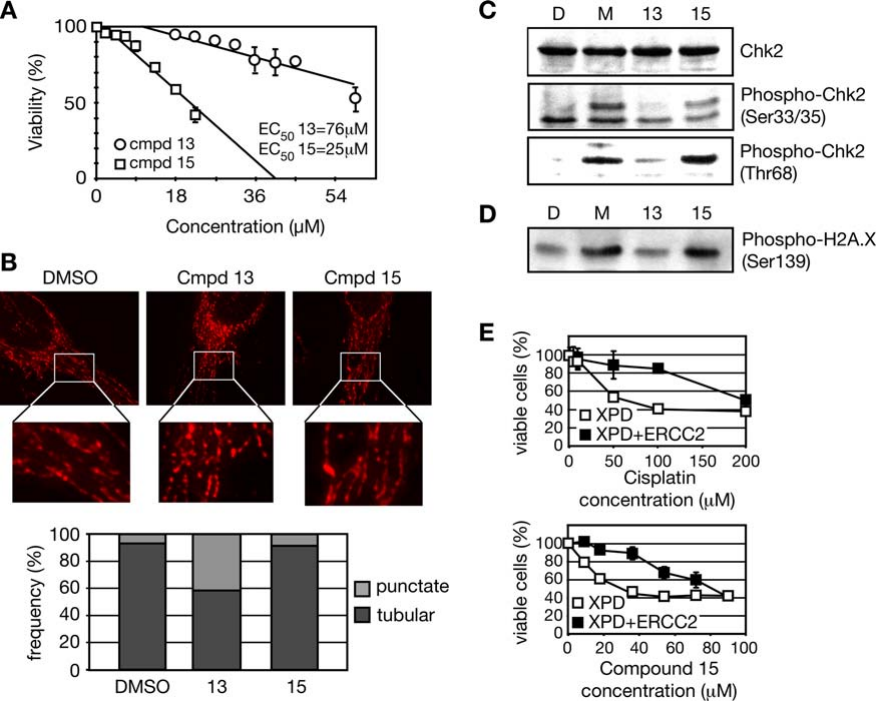
Compound 13 induces mitochondrial fragmentation and compound 15 activates the DNA damage response in mammalian cells. A. Viability of Jurkat cells following treatment with compound 13 or 15. Cells were grown in the presence of increasing concentrations of compounds for two days and viability was measured. B. Mitochondrial morphology of HeLa cells following treatment with compound 13 (45 µM), compound 15 (22.5 µM), or DMSO (0.2%). Mitochondria were stained with anti-ATP synthase (complex V) subunit α antibodies. Deconvoluted optical sections of representative cells are shown (top). The fraction of cells containing punctate, fragmented mitochondria or tubular mitochondria was determined by counting at least 200 cells in each experiment (bottom). C. Immunoblot detection of phosphorylated forms of Chk2. Cells were treated with DMSO (0.2%), MMS (0.0035%), compound 13 (63 µM), or compound 15 (23 µM) for four hours. Cell extracts were run on SDS-PAGE and probed with the respective Chk2 antibodies. D. Immunoblot detection of phosphorylated H2AX. Cells were treated as in (B). Cell extracts were run on SDS-PAGE and probed with anti-phospho-H2AX antibodies. E. Viability of XPD and rescued XPD (XPD+ERCC2) lines following treatment with the indicated concentrations of cisplatin or compound 15.

To determine if the distinct actions of compounds 13 and 15 are evident in mammalian cells, we treated Jurkat cells with increasing concentrations of each compound and measured viability (Figure 6A). Both compounds were toxic to human cells, but the relative cytotoxicity was the reverse of what we observed in yeast, with compound 15 having a lower EC_50_ (25 µM) than compound 13 (76 µM). A similar result was obtained with HeLa cells (data not shown).

We first tested the effect of the two compounds on the mitochondrial morphology of HeLa cells. We treated HeLa cells with DMSO, compound 13, or compound 15 for 4 hours, and observed mitochondrial morphology by indirect immunofluorescent detection of the ATP synthase (complex V) subunit α (Figure 6B). For compound 13 treated cells, we observed a significant increase in the number of cells with punctate, fragmented mitochondria, reminiscent of our observation of fragmented mitochondria in yeast. By contrast, compound 15 treated cells did not show an increase in mitochondrial fragmentation.

We next tested whether either compound caused DNA damage checkpoint activation, a marker for nuclear DNA damage. In mammalian cells, DNA double strand breaks usually result in activation of ATM, whereas ATR is activated in response to UV and stalled replication. ATM is predominantly required for Chk2 activation whereas ATR is predominantly required for Chk1 activation, although there is some cross talk between the pathways (reviewed in [54]–[56]). Jurkat cells were treated with vehicle, with MMS as a positive control, or with compound 13 or 15 (Figure 6C). For cells treated with compound 15, we observed robust phosphorylation of Chk2 with two phospho-specific Chk2 antibodies, indicating the activation of the DNA damage checkpoint. We further tested these cells for phosphorylation of histone H2AX, a chromatin modification that occurs near double strand DNA breaks (reviewed in [57],[58]). Like Chk2 phosphorylation, H2AX phosphorylation was induced by compound 15, but not by compound 13 (Figure 6D).

Finally, we asked whether nucleotide excision repair (NER) was important for tolerance of DNA damage induced by compound 15 in mammalian cells, as was the case in yeast. NER genes are mutant in the repair defective chromosome instability syndrome Xeroderma Pigmentosum (XP) [59],[60]. We utilized a cell line from a patient of the XPD complementation group [61], mutant in the ERCC2 DNA helicase [62], and a control line in which the mutation was restored to the wild type by a transfected ERCC2 expression construct [63], and tested for sensitivity to compound 15, compound 13, and cisplatin. For both cisplatin and compound 15, expression of ERCC2 (XPD+ERCC2) rescued the sensitivity of the NER-defective XPD cell line (XPD; Figure 6E). By contrast, sensitivity to compound 13 was not efficiently rescued by expression of ERCC2 (Figure S4). We conclude that the yeast chemical-genetic profile accurately predicted an important role for NER in the repair of DNA lesions induced by compound 15 in mammalian cells.

Thus our observations in mammalian cells mirror those in yeast: compound 13 causes mitochondrial fragmentation and compound 15 causes damage to the nuclear DNA that is repaired by the NER pathway.

## Discussion

Chemical-genetic profiling in yeast is a robust technique for exploring the mechanism of action of biologically active compounds. Profiling of compound 13 and 15, a nitroso-armed imidazo-pyridine and imidazo-pyrimidine, respectively, suggested that despite the strong likelihood that they cause oxidative stress, they might act in a mechanistically distinct manner. We provide several lines of evidence that compound 13 causes mitochondrial dysfunction, whereas compound 15 causes damage to the nuclear DNA. These different modes of action were also apparent when human cells were treated with compounds 13 and 15, illustrating the utility of chemical-genetic profiling in yeast in predicting mode of action in higher eukaryotes.

Nitroso aromatic compounds are bioactive mainly because they are readily reduced to highly reactive nitro radical anions which activate oxygen [28]. Although there is a possibility that compound 13 and 15 are toxic to cells via other mechanisms, several lines of evidence suggest that compound 13 and 15 are acting as oxidizing agents in vivo. We found that 2-phenylimidazo[1,2-*a*]pyridin-3-amine (compound 151), which is the reduced form of compound 13, was not active on yeast cells (Figure 1B). Furthermore, the antifungal activity of compound 13 and 15 could be partially suppressed by pre-treating cells to induce intracellular accumulation of reduced glutathione (Figure S2), a protective small molecule that is part of the cellular defense against oxidative damage [29],[64]. Finally, we also found that chemical reduction of compounds 13 and 15 in vitro resulted in their inactivation (data not shown).

The chemical-genetic profile of compound 13 was significantly enriched for biological processes such as mitochondrial organization and biogenesis, and oxidative phosphorylation. This profile is reminiscent of that of H_2_O_2_ in which a specific requirement for an intact respiratory chain [37] and a broader requirement for mitochondrial function [38] was observed. Our data also suggest that compound 13 causes mitochondrial dysfunction since cells treated with compound 13 lose their peroxide tolerance, much like those treated with ethidium bromide or the ionophore FCCP. Consistent with this, compound 13 caused dramatic changes in mitochondrial morphology, resulting in extensive mitochondrial fragmentation, a phenotype known to disrupt mitochondrial activity. Maintenance of proper mitochondrial morphology is critical, and fragmentation of mitochondria is an important step in the progression of apoptosis [65].

Compound 15 displayed none of the mitochondria-specific characteristics seen with compound 13. The chemical-genetic profile of compound 15 showed little overlap with that of compound 13, and its profile clearly differed from other oxidizing agents examined. Few DNA repair genes have been identified in genome-wide screens with the oxidants H_2_O_2_, linoleic acid 13-hydroperoxide, menadione, cumene hydroperoxide, and diamide [37],[38]. It has therefore been speculated that loss of viability following treatment with these agents is due to damage to proteins rather than to DNA [37]. Our evidence that compound 15 causes DNA damage suggests that it will be a useful compound in the study of the cellular response to oxidative damage in the nucleus. Importantly, although targeting of oxidative stress to the nucleus has been reported [66], we provide evidence of targeted oxidative stress specifically causing nuclear DNA damage. Although this can be readily inferred from the importance of three DNA repair pathways in compound 15 tolerance, we also demonstrated that compound 15 causes activation of the DNA damage checkpoint and induces mutations in nuclear DNA.

The anti-proliferative and DNA damaging properties of compound 15 are shared by a number of cancer therapeutics, indicating that compound 15 or derivatives of it might be useful in this regard. It is of particular interest that tolerance of compound 15 in mammalian cells required the same DNA repair pathway, nucleotide excision repair, as was found in yeast. DNA repair pathway-specific toxicity affords the possibility of rational therapeutic approaches based on targeting cells defective in a given pathway. Additionally, synergy might be obtained by targeting multiple DNA repair pathways independently. Mitochondria are also an attractive anti-cancer target, as pharmacological modulation of mitochondrial permeability can result in apoptotic cell death [67]. The mitochondrial fragmentation caused by compound 13 might reflect induction of the permeability transition pore, as has been observed with other forms of oxidative stress [68]. Ongoing studies to determine the precise mechanism of action of compound 13 will reveal its potential as a therapeutic.

## Materials and Methods

### Yeast Strains

The yeast strain BY4741 [69] was used in the growth rate study. Gene deletion mutant strains [3] are available from Open Biosystems. Standard yeast media and growth conditions were used [70]. The rho^0^ strain lacking mitochondrial DNA was generated from BY4741 as described [71].

### Compound Chemistry

Compounds **2**, **3**, **16** and **18** were synthesized by condensation of the non-substituted 2-amino-pyridine (**1a**) or 2-amino-pyrimidine (**1b**) intermediate with the chlorinated precursors (Cl-CH_2_-CO-R; R = Ph, p-MeO-Ph, CO_2_-Et), as reported [72]–[75]. 3-formyl-2-(4-methoxyphenyl)imidazo[1,2-*a*]pyrimidine (**14**) and 3-formyl-2-phenylimidazo[1,2-*a*]pyridine (**17**) were generated from compounds **3** and **2** respectively as described [24]. Treatment of **2** and **18** with sodium nitrite in acetic acid under ambient conditions, as reported [21], gave **13** and **15** respectively with an overall yield of 70–80%. The reduction of the nitroso group at position 3 of **13** was carried out with tin in HBr medium, providing **151** with a yield of 90%. Acetylation of the amine group of **151** by acetic anhydride gave a novel compound (**152**) bearing an amide group at position 3. Further details are presented in the Supplementary Methods (Text S1).

### Growth and Viability Analysis

For the growth analysis the different compounds were added to logarithmic phase cells and growth rate was monitored by measuring the optical density of cells (OD600) as a function of time (hours) in rich medium. All compounds were diluted in 100% DMSO, and all assays, including the “no compound” control, contained 1% DMSO.

For viability analysis, cells were grown in YPD [76] in the presence of 0 to 15.6 µM compound 13, or 0 to 107 µM compound 15, for 2 hours at 30°C. Cells were then spread on YPD plates to determine the number of colony-forming units.

### Chemical–Genetic Screens

Screens of the homozygous deletion pool, the heterozygous deletion pool, and the haploid deletion pool were performed as described [10],[11], with 9 µM of compound 13 or compound 15, which corresponded to 10% growth inhibition of the wild type diploid. Fitness defect scores, based on a tag specific algorithm that takes into account the intensities of each tag in the compound treated cells compared to non-treated cells, were calculated for each deletion strain in the pool for each experiment as described [9]. The complete scores for the screens with compounds 13 and 15 are available at http://biochemistry.utoronto.ca/brown/data.html. Microarray data have been deposited in the ArrayExpress database (Accession numbers E-TABM-560 and A-MEXP-1420). Two-dimensional hierarchical clustering was performed as described [10]. Enrichment of GO terms was calculated from the hypergeometric distribution. P indicates the probability that each GO term would be expected by chance to occur at equal to or greater than the observed frequency within an identified set of genes, given the known frequency of occurrence among the genes represented in the heterozygous and homozygous deletion mutant pools. GO biological processes were filtered for processes containing >400 or <20 genes, to eliminate processes that are too general or too specific.

### Peroxide Response and Halo Assays

For peroxide adaptation experiments, log phase cells were grown in YPD or YPD containing sub-lethal (0.25 mM) concentration of H_2_O_2_ for one hour before treatment with higher concentrations of H_2_O_2_ (0 to 3.0 mM, in 1.25-fold increments), compound 13 (0 to 13.4 µM, in 1.25-fold increments), or compound 15 (0 to 44.6 µM, in 1.25-fold increments). Optical density was monitored for 24 hours. The adaptive response for each concentration of compound was calculated by dividing the time it takes for non pre-treated (or pre-treated) cells in the presence of compound to reach an OD_600_ of 0.15 by the time it takes the same cells in the absence of the compound to reach the same OD.

Peroxide sensitivity was determined using halo assays. Wild type cells were grown in the presence of 1% DMSO, 9 µM (1 EC_50_) compound 13, 56 µM (1 EC_50_) compound 15, 25 µM ethidium bromide, 1% MeOH (the vehicle for FCCP), or 15.74 µM FCCP for 24 hours. Equal numbers of treated cells were spread onto YPD plates and 8 mm Whatman disks containing 6% H_2_O_2_ were put onto the middle of each plate. Plates were incubated at 30°C for 2 days and then photographed. The diameter of the growth inhibition was measured using OpenLab (ImproVision, Waltham, MA), the area of inhibition was calculated, and plotted as the average of two independent experiments.

### Mitochondrial Morphology

Yeast mitochondria were visualized by transforming cells with pVT100U-mtGFP [77] encoding mitochondria-targeted GFP. Cells were treated with DMSO (1%), compound 13 (9 µM), or compound 15 (56 µM) for three hours, and mitochondrial morphology was observed by taking Z-sections of GFP fluorescence with an Imager Z1 Zeiss microscope using a 63× objective and deconvolution with ImproVision Velocity software. At least three hundred cells of each compound treated sample were counted and grouped either into normal tubular morphology, or punctate morphology.

HeLa cell mitochondrial morphology was observed by indirect immunofluorescence of the ATP synthase (complex V) subunit α using CV-α antibody (Mitosciences, Oregon) as described by the supplier, and quantified as described above.

### Mutation Rate Analysis

Cells were treated with 1% DMSO, 0.035% MMS, 9 µM (1 EC_50_) compound 13, or 56 µM (1 EC_50_) compound 15 for 2 hours, and allowed to grow to saturation. Cells were plated on complete and selective media to determine the frequency of mutations in the *CAN1* gene, as described previously [78]. Mutation rates were calculated using the method of the median [79], and are averages of three fluctuation tests of 7 independent assays.

### Immunoblotting

Wild type yeast, or DNA repair mutants, were treated with 1% DMSO, 0.035% MMS, 9 µM (1 EC_50_) compound 13, or 56 µM (1 EC_50_) compound 15 for 2 hrs. 1×10^8^ cells were TCA fixed, collected, and used for Rad53 immunoblots, as described [80]. Rad53 was detected with yC-19 (Santa Cruz Biotechnology). Blots were re-probed for Tub1 (YOL1/34; Novus Biologicals) as a loading control.

Jurkat cells were grown in 0.2% DMSO, 0.0035%MMS, 63 µM compound 13, or 23 µM compound 15 for 4 hours, washed with 1× PBS, and extracts were made by lysing the cells in SDS loading dye. Rabbit polyclonal antibodies against Chk2, Phospho-Chk2 (Thr 68), and Phospho-Chk2 (Ser 33/35) were from Cell Signaling Technology. Antibodies against phosphorylated H2AX (Ser139; clone JBW301) were from Millipore.

### Mammalian Cells

Details of the growth and maintenance of Jurkat cells (ATCC), HeLa cells (ATCC), and XPD lines GM08207 and GM15877 (Coriell Institute for Medical Research) are described in the Supplementary Methods (Text S1).

## Supporting Information

Figure S1 Yeast bioactivity of imidazo-pyridine and imidazo-pyrimidine compounds. Cultures of *S. cerevisiae* were grown in the presence of 100 µg/ml of the indicated compounds (except for compound 13, included as a control at 20 µg/ml) and their optical density was measured and compared to the untreated (DMSO only) control at 4, 8, and 20 hours.
(0.05 MB PDF)

Figure S2 Induction of intracellular GSH increases tolerance of compound 13 and 15. Growth of cells treated with DMSO (red line), compounds (black line), or cells with increased intracellular GSH in the presence of compounds (blue line) were monitored as a function of time.
(0.08 MB PDF)

Figure S3 Compound 15 causes Rad53 activation in vivo. Cells were treated with increasing amounts of either compound 13 or 15 and fixed with 10% TCA. Cell extracts were fractionated on SDS-PAGE, and Rad53 protein detected by immunoblot analysis. The positions of Rad53 and the activated, phosphorylated form of Rad53 (Rad53-P) are indicated. The immunoblots were re-probed to detect tubulin (tub), as a loading control. Compound 15 activated Rad53 at concentrations of 45 µM and 67.5 µM (0.8 EC^50^ and 1.6 EC^50^). By contrast, compound 13 did not induce detectable Rad53 phosphorylation, even at 112.5 µM (12.5 EC^50^).
(0.23 MB PDF)

Figure S4 ERCC2 expression does not significantly rescue sensitivity of XPD cells to compound 13. Viability of XPD and rescued XPD (XPD+ERCC2) lines was determined following treatment with the indicated concentrations of compound 13.
(0.05 MB PDF)

Text S1 Supplemental methods.
(0.05 MB DOC)
