# Supplementary material for: Chemical–Genetic Profiling of Imidazo[1,2-a]pyridines and -Pyrimidines Reveals Target Pathways Conserved between Yeast and Human Cells
Source: PLoS Genet. 2008 Nov 28;4(11):e1000284. doi: 10.1371/journal.pgen.1000284 (PMC2583946; doi:10.1371/journal.pgen.1000284)
Supplement: Figure S1 — Yeast bioactivity of imidazo-pyridine and imidazo-pyrimidine compounds. Cultures of S. cerevisiae were grown in the presence of 100 µg/ml of the indicated compounds (except for compound 13, included as a control at 20 µg/ml) and their optical density was measured and compared to the untreated (DMSO only) control at 4, 8, and 20 hours. (0.05 MB PDF) [file pgen.1000284.s001.pdf]

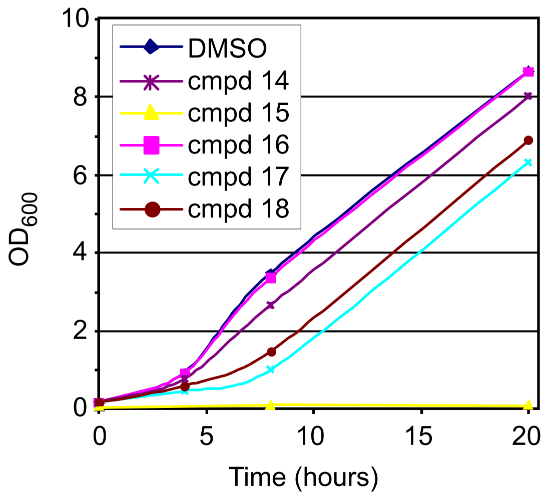

Figure S1. Yeast bioactivity of imidazo-pyridine and imidazo-pyrimidine compounds. Cultures of S. cerevisiae were grown in the presence of 100 μg/ml of the indicated compounds (except for compound 13, included as a control at 20 μg/ml) and their optical density was measured and compared to the untreated (DMSO only) control at 4, 8, and 20 hours.
