# Supplementary material for: Chemical–Genetic Profiling of Imidazo[1,2-a]pyridines and -Pyrimidines Reveals Target Pathways Conserved between Yeast and Human Cells
Source: PLoS Genet. 2008 Nov 28;4(11):e1000284. doi: 10.1371/journal.pgen.1000284 (PMC2583946; doi:10.1371/journal.pgen.1000284)
Supplement: Figure S2 — Induction of intracellular GSH increases tolerance of compound 13 and 15. Growth of cells treated with DMSO (red line), compounds (black line), or cells with increased intracellular GSH in the presence of compounds (blue line) were monitored as a function of time. (0.08 MB PDF) [file pgen.1000284.s002.pdf]

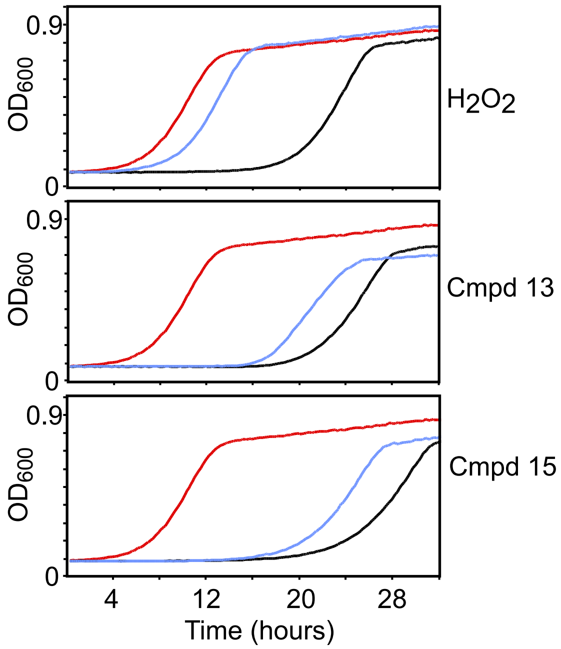


**Figure S2.** Induction of intracellular GSH increases tolerance of compound 13 and 15. Growth of cells treated with DMSO (red line), compounds (black line), or cells with increased intracellular GSH in the presence of compounds (blue line) were monitored as a function of time.
