# Supplementary material for: Chemical–Genetic Profiling of Imidazo[1,2-a]pyridines and -Pyrimidines Reveals Target Pathways Conserved between Yeast and Human Cells
Source: PLoS Genet. 2008 Nov 28;4(11):e1000284. doi: 10.1371/journal.pgen.1000284 (PMC2583946; doi:10.1371/journal.pgen.1000284)
Supplement: Figure S3 — Compound 15 causes Rad53 activation in vivo. Cells were treated with increasing amounts of either compound 13 or 15 and fixed with 10% TCA. Cell extracts were fractionated on SDS-PAGE, and Rad53 protein detected by immunoblot analysis. The positions of Rad53 and the activated, phosphorylated form of Rad53 (Rad53-P) are indicated. The immunoblots were re-probed to detect tubulin (tub), as a loading control. Compound 15 activated Rad53 at concentrations of 45 µM and 67.5 µM (0.8 EC50 and 1.6 EC50). By contrast, compound 13 did not induce detectable Rad53 phosphorylation, even at 112.5 µM (12.5 EC50). (0.23 MB PDF) [file pgen.1000284.s003.pdf]

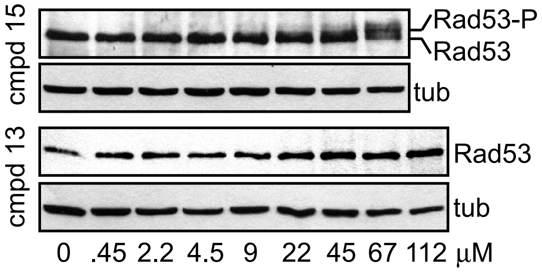

Figure S3. Compound 15 causes Rad53 activation in vivo. Cells were treated with increasing amounts of either compound 13 or 15 and fixed with 10% TCA. Cell extracts were fractionated on SDS-PAGE, and Rad53 protein detected by immunoblot analysis. The positions of Rad53 and the activated, phosphorylated form of Rad53 (Rad53-P) are indicated. The immunoblots were re-probed to detect tubulin (tub), as a loading control. Compound 15 activated Rad53 at concentrations of 45 μM and 67.5 μM (0.8 EC_{50} and 1.6 EC_{50}). By contrast, compound 13 did not induce detectable Rad53 phosphorylation, even at 112.5 μM (12.5 EC_{50}).
