# Supplementary material for: Chemical–Genetic Profiling of Imidazo[1,2-a]pyridines and -Pyrimidines Reveals Target Pathways Conserved between Yeast and Human Cells
Source: PLoS Genet. 2008 Nov 28;4(11):e1000284. doi: 10.1371/journal.pgen.1000284 (PMC2583946; doi:10.1371/journal.pgen.1000284)
Supplement: Figure S4 — ERCC2 expression does not significantly rescue sensitivity of XPD cells to compound 13. Viability of XPD and rescued XPD (XPD+ERCC2) lines was determined following treatment with the indicated concentrations of compound 13. (0.05 MB PDF) [file pgen.1000284.s004.pdf]

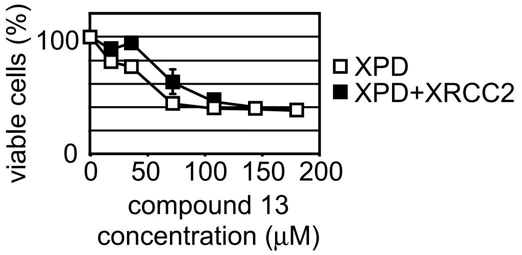

Figure S4. ERCC2 expression does not significantly rescue sensitivity of XPD cells to compound 13. Viability of XPD and rescued XPD (XPD+ERCC2) lines was determined following treatment with the indicated concentrations of compound 13.
