# Supplementary material for: Chemical–Genetic Profiling of Imidazo[1,2-a]pyridines and -Pyrimidines Reveals Target Pathways Conserved between Yeast and Human Cells
Source: PLoS Genet. 2008 Nov 28;4(11):e1000284. doi: 10.1371/journal.pgen.1000284 (PMC2583946; doi:10.1371/journal.pgen.1000284)
Supplement: Text S1 — Supplemental methods. (0.05 MB DOC) [file pgen.1000284.s005.doc]

## Text S1. Supplemental Methods

##### Chemical synthesis of imidazo-pyridine and imidazo-pyrimidine compounds

Melting points were determined using a Totoli capillary apparatus and are uncorrected. ^1^H NMR spectra were recorded on a Bruker AC 100, AC 250 or AM 400 WB spectrometer. Mass Spectra were obtained on a LKB 2091 spectrometer by electronic impact.

Synthesis of 2-phenyl-3-nitroso-imidazo[1,2-a]pyridine (**13**): compound **13**, green powder, was obtained from compound **2** as described previously [1]. M. p. = 165-167 °C; ^1^H NMR (200.134 MHz, CDCl_3_) δ (ppm): 9.92 (dd, 1H, H_5_), 8.41 (dd, 1H, H_8_), 8.22 (s, HPh), 7.78 (m, 2HPh), 7.41 (pst, 1 H, H_7_), 7.12 (m, HPh), 7.08 (pst, H_6_).

Synthesis of 2-phenyl-3-nitroso-imidazo[1,2-a]pyrimidine (**15**): compound **15**, green powder, was obtained from compound **18** as described previously [1]. M. p. = 153-154°C; IR (KBr, cm^-1^): 1580 (N=O); ^1^H NMR (CDCl_3_, 300 MHz), δ: 10.00 – 9.97 (dd, 1H, J H_5_-H_6_ = 6.60 Hz, J H_5_-H_7_ = 2.10 Hz, H_5_) ; 8.94 – 8.91 (dd, 1H, J H_7_-H_6_ = 4.50 Hz, J H_7_-H_5_ = 2.10 Hz, H_7_) ; 8.77 – 8.75 (dd, 1H, J H_6_-H_5_ = 6.90 Hz, J H_5_-H_7_ = 1.50 Hz, H_6_) ; 7.63 – 7.51 (m, 4H, H_10_, H_11_, H_13_, H_14_) ; 7.30 – 7.27 (dd, 1H, J H_12_-H_11_ = 6.60 Hz, J H_12_-H_10_ = 4.50 Hz, H_12_); ^13^ C NMR (CDCl_3_, 75 MHz), δ: 158.8 (C_8a_); 134.1 (C_3_); 132.8 (C_5_); 131.8 (C_7_); 131.4 (C_2_); 129.4 (C_6_); 115.7 (C Ph); MS (EI, m / z): 224 (M^+^) / 100%; 194 (M^+^ - N=O) / 35%; 147 (M^+^ - C_6_H_5_) / 10%; 117 (M^+^ - [N=O] – [C_6_H_5_] ) / 12%.

Synthesis of 2-phenylimidazo[1,2-a]pyridin-3-amine (**151)**: 1 g (4,2 mmol) of compound **13** was added portions-wise, during 30 min, to a suspension of tin (1 g) in frozen HBr (30 ml). After 3 h of stirring at ambient temperature, the precipitate was filtered, diluted with water (30 ml), and then alkalized with NH_4_OH. The solid obtained was extracted overnight by CH_2_Cl_2_ in a Soxhlet apparatus. After drying (Na_2_so_4_), CH_2_Cl_2_ is evaporated under vacuum to give compound 151 as a yellowish powder (yield 80 %). M. p. = 210 - 212°C; IR (KBr, cm^-1^): 3400, 1630, 750 (NH_2_) ; ^1^H NMR (CDCl_3_, 90 MHz), δ : 3,80 (NH_2_), 7,15 (t, 1 H), 7.46 (m, 5 H), 8,10 (m, 2 H), 8,46 (m, 1 H). MS (EI, m / z): 209 (M^+^) / 100%.

Synthesis of N-(2-phenylimidazo[1,2-a]pyridin-3-yl)acetamide (**152***)***:** compound **151** (0.50 g) in toluene (10 ml) is treated with acetic anhydride (0,3 ml). After stirring for two hours, toluene was eliminated under reduced pressure and the residue was washed with water, and then dried to afford 0.45 g of compound **152** as a colorless powder. ^1^H NMR (CDCl_3_, 300,14 MHz), δ : 2,23 (s, 3H, CH_3_), 6.95 (m, 1H, H_6_), 7,28 - 762 (m, 5H), 7,99 (m, 2H), 8,08 (dd, 1H, H_5_), 10,17 (s, NH).

*Glutathione induction.* The rationale for induction of glutathione was as described [2,3]. Log phase cells were either grown in YPD or GSH media [0.5 M glucose, 0.01 M MgCl_2_, 0.02 M L-glutamate, 0.02 M L-cysteine, 0.02 M glycine, and 0.1 M potassium phosphate buffer (pH7.4)] for one hour. Cells were pelleted, resuspended in YPD and treated with H_2_O_2_ (2 mM), compound 13 (9 μM) or compound 15 (29 μM) at concentrations which inhibited the growth of WT cells to approximately the same extent. Optical density was measured every 15 minutes with a GENios microplate reader (Tecan, Durham, North Carolina, United States) for 24 hours.

*Mammalian cell culture.* Jurkat cells were obtained from ATCC and grown at 37°C in a humidified atmosphere of 5% CO_2_. Jurkat cells were maintained in RPMI 1640 medium supplemented with 10% heat-inactivated fetal bovine serum albumin (FBS), 1mM sodium pyruvate, and 2mM L-glutamine. To determine viability, Jurkat cells (5 x 10^5^ cells/ml) were grown in the presence of 0 to 67 µM compound 13 or 0 to 22 µM compound 15 for two days. Cells were then stained with Guava ViaCount Reagent (Guava Technologies, Hayward, CA), and cell viability were determined based on differential permeability of DNA-binding dyes with a Guava flow cytometer (Guava Technologies, Hayward, CA). EC_50_ was determined as the concentration at which 50% of cells were dead after two days of growth.

HeLa cells were obtained from ATCC and grown at 37°C in a humidified atmosphere of 5% CO_2_. HeLa cells were maintained in Eagle Minimum Essential Medium supplemented with 10% FBS, 1mM non-essential amino acids, and 10mM sodium pyruvate. HeLa cells were allowed to adhere to coverslips overnight, and treated with 0.5% DMSO, or equitoxic concentrations of compound 13 (45 µM) or compound 15 (22.5 µM) for 4 hours.

The cells lines GM08207 and GM15877 were obtained from Coriell Institute for Medical Research and maintained in DMEM (Gibco) with 2mM L-glutamine and 10% heat inactivated FBS at 37^o^C with 5% CO_2_. The corrected phenotype of GM15877 was selected by supplementing the growth media with 0.6 mg/ml Geneticin. Viability of cells in the presence of cisplatin (0 μM, 1 μM, 5 μM, 10 μM, 50 μM, 100 μM, and 200 μM), compound 15 (0 μM, 9 μM, 18 μM, 36μM, 54 μM, 72 μM, and 90 μM), and compound 13 (0 μM, 18 μM, 36 μM, 72 μM, 108 μM, 144 μM, and 180 μM) was measured with the Alamar blue assay (Invitrogen). For this assay, 16000 cells/250 μl were seeded into 96 well plates and allowed to attach overnight. The concentration of cells were chosen such that cultures reached ~80% confluence after 3 days incubation. Compounds were then added to cells and incubated for 48 hours. One-tenth volume (25 μl) of Alamar blue was added to each well after compound incubation, mixed, and incubated at 37^o^C for 6 hours until ~80% of the Alamar blue was reduced. Absorbance was measured at 570 nm and 600 nm and cell viability was calculated as described by the manufacturer.

**References:**

1. Rival Y, Grassy G, Tandou A, Escalle R (1991) Antifungal activity in vitro of some imidazo[1,2-a]pyrimidine derivatives. Eur J Med Chem 26: 13-18.

2. Izawa S, Inoue Y, Kimura A (1995) Oxidative stress response in yeast: effect of glutathione on adaptation to hydrogen peroxide stress in Saccharomyces cerevisiae. FEBS Lett 368: 73-76.

3. Murata K, Tani K, Kato J, Chibata I (1980) Continuous production of glutathione using immobilized microbial cells containing ATP generating system. Biochimie 62: 347-352.
